# Disablement Pensions and Reduced Sickness Benefit

**Published:** 1915-07-17

**Authors:** 


### NATIONAL INSURANCE ACT DEVELOPMENTS.

### Disablement Pensions and Reduced Sickness Benefit.

In a recent circular (A. S. 163) issued by the Insurance Commissioners attention is drawn to the provisions of the National Insurance (Part I.) Amendment Act, 1915, and the procedure entailed by the new Act is explained in detail. The circular is important because it makes clear several points in the new Act which were somewhat obscure.

It will be remembered that the new Act provides, among other things, that an approved society shall be relieved of its liability to pay sickness or disablement benefit to the extent of 5s. a week in respect of a member who, upon his discharge from the Navy or Army, is granted a disablement pension. This arrangement was made in view of the increases in the rates of pension which, on the recommendation of a Select Committee, received Parliamentary sanction this spring. It is now pointed out that the reduction in benefit only applies to those men who obtain the *total* disablement pension in consequence of the present war, and not to those who obtain pension for partial disablement, and, moreover, the rate of benefit is not affected in any way by normal pensions. Again, the reduction of benefit is only to continue so long as the total disablement pension is being received by the member, and it is pointed out that as the total and partial disablement pensions are reviewed, generally after a year, there may be cases where a society which has been relieved of the payment of 5s. a week will have to resume the payment of full rate of benefit upon the revision of pension. Similarly a partial disablement pension may be converted into a total disablement pension, and consequently a society, although not obtaining relief at once, may do so ultimately.

The reduction of benefit, it will be remembered, is to date back to the date at which the pension is granted, or to March 1, 1915, in the case of men discharged before that date. It is not, however, necessary for a society to defer payment of benefit pending the decision as to the grant of pension. It can pay at the full rate of benefit—sickness or disablement, as the case may be—and make good the deficiency subsequently. Somewhat elaborate arrangements are made whereby approved societies may obtain the refund of benefits paid in excess of their true liability pending the decision of the Admiralty or War Office as to the grant of a total disablement pension. In the normal case, however, as the member will usually be in receipt of a lump sum, representing the arrears of his pension, it is suggested that the best course will be for a society to stop all payments at once until the amount overpaid has been recovered. In certain cases, where this method would not provide the means for a full refund, the Admiralty or War Office will, as far as possible, retain out of the arrears of pension an amount sufficient to recoup the society for the advances of benefit.

Perhaps the most important feature of the circular is the recognition which it gives to co-operation between the different Government Departments, by the provision of appropriate forms upon which notification is to be sent to the Insurance Commissioners of the grant of total disablement pension when it has been impossible to notify the approved society to which the discharged sailor or soldier belongs. This makes us hope that it will still be possible for the same Government Departments to co-operate with regard to the provision of medical treatment for soldiers on leave, at their own homes.

The recent development in the organisation of trades, for the purpose of the better carrying out of the war, has led to the issue of further regulations by the Insurance Commissioners in regard to the payment of contributions by the men affected. The contents of these regulations are explained in a circular (A. S. 169), and it will at once be seen that the matter is one of considerable interest.

Two classes of persons are covered by the regulations—namely, (1) those who have enlisted in the "Dock Battalions" at Liverpool, and (2) those who have been, or may in future be, sent back by the Army authorities to work in engineering or other manufactories. Such men are in receipt of wages in addition to their Army pay, and consequently, although they are in the Forces of the Crown, the new regulations require that the full normal rates of contribution shall be paid. On the other hand, the men in question will be entitled to the full normal benefits from the insurance, as against the restricted benefits to which Navy and Army members are ordinarily entitled.

If at some future time a man so serving in civil employment returns to service with the Forces his status as regards the insurance will revert to the normal condition applicable to men in the Forces.

